# *Plasmodium malariae* Detected by Microscopy in the International Bordering Area of Mizoram, a Northeastern State of India

**DOI:** 10.3390/diagnostics12082015

**Published:** 2022-08-20

**Authors:** Kuldeep Singh, Praveen Kumar Bharti, Naorem Chaoba Devi, Naseem Ahmed, Amit Sharma

**Affiliations:** 1 ICMR—National Institute of Malaria Research, Field Station, Guwahati 781005, India; 2 ICMR—National Institute of Malaria Research, New Delhi 110077, India; 3 International Centre for Genetic Engineering and Biotechnology, New Delhi 110067, India

**Keywords:** *Plasmodium malariae*, *Plasmodium falciparum*, *Plasmodium vivax*, RDTs, malaria, microscopy, polymerase chain reaction

## Abstract

Northeastern states of India share international borders with Myanmar, China, Bangladesh, and Bhutan, contributing 7.45% of the overall malaria cases in the country. Mizoram accounts for the highest malaria burden in the northeastern states, with perennial transmission in the hilly and deep-forested areas. *Plasmodium falciparum* (93%) is the most prevalent human *Plasmodium* species, followed by *P. vivax*; however, information on *P. ovale* and *P. malariae* is negligible. Rapid diagnostic tests (RDTs) are the most preferred malaria diagnostic tool followed by microscopy in this high malaria-endemic region. The present epidemiological study was carried out in July and August 2019 to assess the malaria burden in and around the Chawngte primary health center, Lawngtlai District of Mizoram, using RDTs and microscopy as diagnostic tools. World Health Organization-certified level I microscopists examined the blood smears. Diagnosis using RDTs resulted in 151 malaria cases (*P. falciparum*: 136; *P. vivax*: 15) out of 948 screened fever cases. However, blood smear examination detected 179 cases (*P. falciparum*: 154; *P. vivax*: 17; mixed *P. falciparum* + *P. vivax* infection: 3; *P. malariae*: 5). Analysis revealed that the risk of malaria infection was higher in the ≥5-year-old subjects than in the under-5 age group. The mean parasite density of *P. malariae* (1455.00/μL blood) was the lowest; cf. with *P. falciparum*: 12,275.08/μL blood. Surveillance at the point-of-care level using microscopy was able to detect all the four human *Plasmodium* species and their mixed infections, including *P. malariae*, which were missed with RDTs. Thus, the quality of microscopy along with trained manpower should be strengthened to diagnose all human malaria parasite species (particularly *P. malariae* and *P. ovale*) until the molecular tools are deployed at the field level to achieve malaria elimination by 2030.

## 1. Introduction

India accounted for 83% of the estimated malaria cases reported from the World Health Organization (WHO) South-East Asia Region in 2020 [1]. India has varied transmission intensities across its landscape and high malaria endemic states in the center, east, and northeast, contributing 80% of the malaria cases [2]. Northeastern states of India contributed 7.45% of the total malaria cases in 2019. Mizoram alone contributes 2.52% of the overall malaria cases in the country and almost one-third of the patients from northeastern India. *Plasmodium falciparum* (93.76%) is the most prevalent malaria infection, having perennial transmission in the hilly and deep-forested area of Mizoram. Among 11 districts of the state, Lawngtlai, Lunglei, and Mamit have an annual parasite index (API) of above 10. In 2021, Lawngtlai District reported 2238 positive malaria cases and three deaths due to malaria [3]. 

Besides *P. falciparum* and *P. vivax*, limited data on regional and global transmission patterns of *P. malariae*, *P. ovale*, and *P. knowlesi* are available [4,5,6,7]. Diagnosis and treatment of non-*P. falciparum/P. vivax* species should also be focused on achieving the malaria elimination target [4,8,9]. 

In the early phase of malaria elimination, the focus on identifying and quantifying *Plasmodium* species can help better implement diagnosis strategies and treatment guidelines [10]. Accurate malaria diagnosis is essential for effective disease management. Epy World Health Organization (WHO) recommends universal testing of all suspected malaria cases before initiation of treatment. Rapid diagnostic tests (RDTs) have revolutionized malaria control efforts in the endemic regions where adequate microscopy facilities are unavailable [11]. RDTs may have variable consistency, quality control, and performance capabilities at the temporal and spatial levels [12]. Deletion of the histidine-rich protein 2 (HRP2) gene in *P. falciparum* results in false-negative RDTs, and a reduction in sensitivity owing to low parasitemia may complicate the diagnosis at the field level [13]. Polymerase chain reaction (PCR)-based diagnosis is the most reliable tool having high sensitivity, high specificity, and capability of diagnosing both human and zoonotic *Plasmodium* species [14,15]. Tahar et al. (1997) developed an alternative PCR method for identifying *P. malariae* using the circumsporozoite protein gene as the DNA target. It was reported to be a suitable add-on in clinical diagnosis and feasible for mass screening in the community [16]. Recent advances in molecular diagnostic tools are the most promising in detecting and quantifying human and zoonotic *Plasmodium* species, their mixed infections, and submicroscopic parasitemia in the community.

However, the need for advanced molecular biology laboratories and skilled biologists limits its application in rugged and inaccessible areas [17]. Microscopic diagnosis of the malaria parasite is a gold-standard technique used since the identification of malaria parasites. Malaria microscopy is easy to use and provides additional information on the gametocyte load in the community, which is critical for malaria transmission. Microscopic diagnosis has great importance in clinical research on the prevalence of non-*P. falciparum*/non-*P. vivax* malaria and therapeutic efficacy of antimalarials. However, it is a labor-intensive technique that requires skilled human resources, unable to detect submicroscopic objects and differentiate between recrudescence and reinfection of malaria. *P. malariae* and their mixed infections have been reported from other northeastern states and central India. In this region, *Anopheles baimaii* and *An. minimus* are evidenced as the primary vectors transmitting human malaria [18]. However, the distribution of these species varies considerably throughout the region [18,19]. Therefore, the present study investigated the prevalence of all human malaria parasites, including *P. malariae* infection, using microscopy from the high endemic district of Mizoram, a northeastern state, in terms of malaria elimination. 

## 2. Materials and Methods

### 2.1. Description of the Study Area

**Figure 1:**
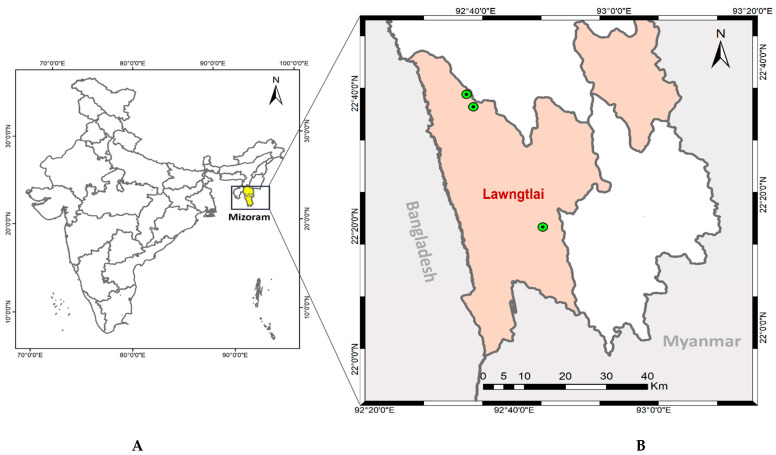
Map showing study sites (**A**) Mizoram, India; (**B**) the green circle shows the location of the *P. malariae*-positive cases detected in Lawngtlai District of Mizoram (India).

Mizoram is one of the northeastern states of India, bordering Bangladesh and Myanmar, having a hilly terrain and inaccessible areas [20]. Lawngtlai District is located along the Myanmar and Bangladesh border, with a thick virgin forest and a hilly terrain (Figure 1). Study site “Chawngte” (22°37′10″ N, 92°38′11″ E) has a population of 45,307 and is situated in the western part of Lawngtlai District, which shares an international border with Bangladesh.

### 2.2. Study Population and Malaria Diagnosis

Active fever surveys were conducted in July–August 2019 using bivalent RDTs and microscopy to diagnose malaria parasites. After the written consent/assent, thick and thin blood smears were prepared per the method reported elsewhere. The specimens were stained (Giemsa stain) to give the parasites a distinctive appearance. The thick and thin smears were examined with a 100× oil immersion objective. Two WHO-certified microscopists examined the smears at the field level. The third independent microscopist further assessed the thick and thin blood smears for quality control purposes. A blood smear was declared positive only after the concordant findings of two independent microscopists. The positive malaria cases were treated as per the National Drug Policy on Malaria (2013) [21].

### 2.3. Estimation of Parasitemia

Parasite density per microliter of blood was estimated by counting the number of asexual parasites per 200 (500 in case of low parasitemia) leukocytes in a thick blood smear using Equation (1). 


(1) $$\text{Parasite density per}\;\mu L=\frac{\text{Number of parasite count}}{\text{Number of leucocyte count}}\times (6000)$$

### 2.4. Quality Control

For quality control, another expert microscopist (WHO level 1), independently and blinded to previous microscopic findings, reassessed 100% of the positive cases and 10% of the negative smears. 

### 2.5. Data Analysis

Statistical data analysis was performed using software package SPSS v.20.0.

## 3. Results

**Figure 2:**
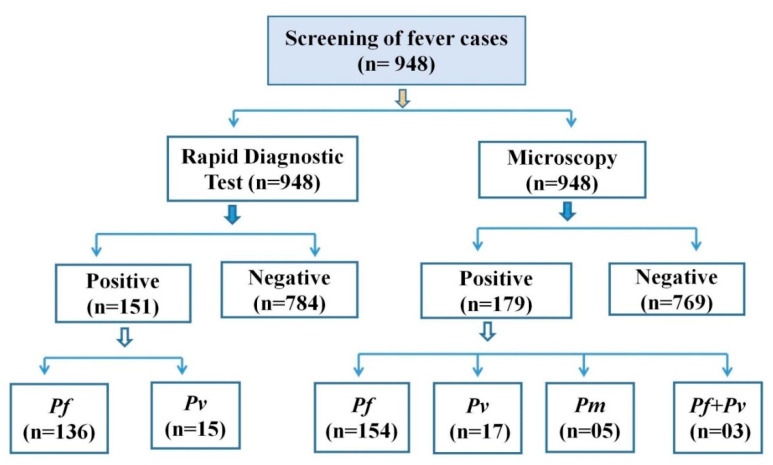
Screening of the fever cases and malaria positivity at the study site (*Pf = P. falciparum*; *Pv = P. vivax*; *Pm = P. malariae*).

A total of 948 cases were screened for malaria parasites using RDTs and microscopy, of which 151 were positive as per RDTs and 179 were positive as per microscopy. Using RDTs, 14.34% were detected as *P. falciparum*, 1.58%—as *P. vivax*. However, in the microscopic examination, the positivity of *P. falciparum* and *P. vivax* was found to be 16.24% and 1.79%, respectively. Further, mixed infection (*P. vivax* + *P. falciparum*) was found in three patients (0.32%), and *P. malariae* was detected in five cases (0.53%) from the study population. All the positive cases diagnosed with RDTs were also found to be positive by malaria microscopy. The results are summarized in Figure 2. 

**Figure 3:**
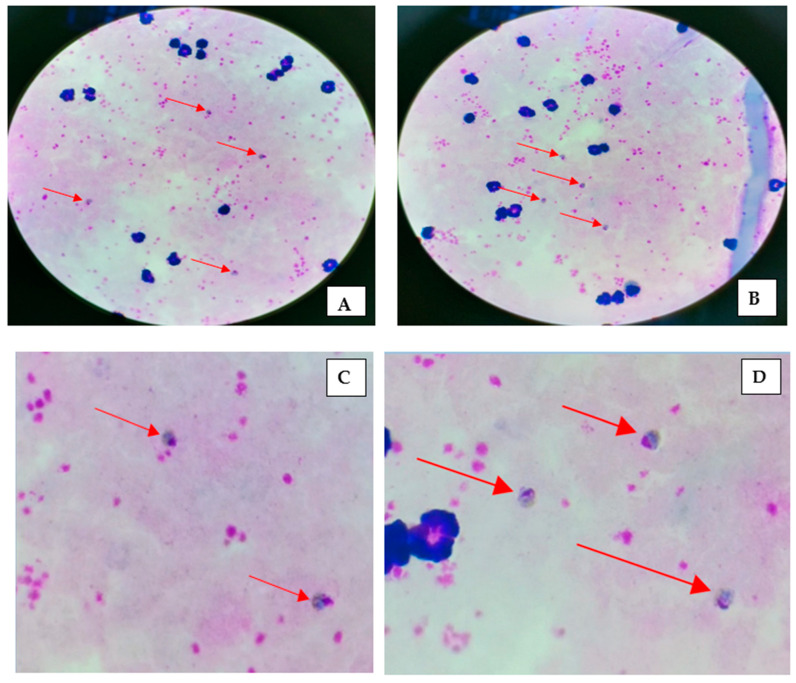
Thick smear showing *Plasmodium malariae* trophozoites (**A**,**B**) and their respective magnified images (**C**,**D**).

**Figure 4:**
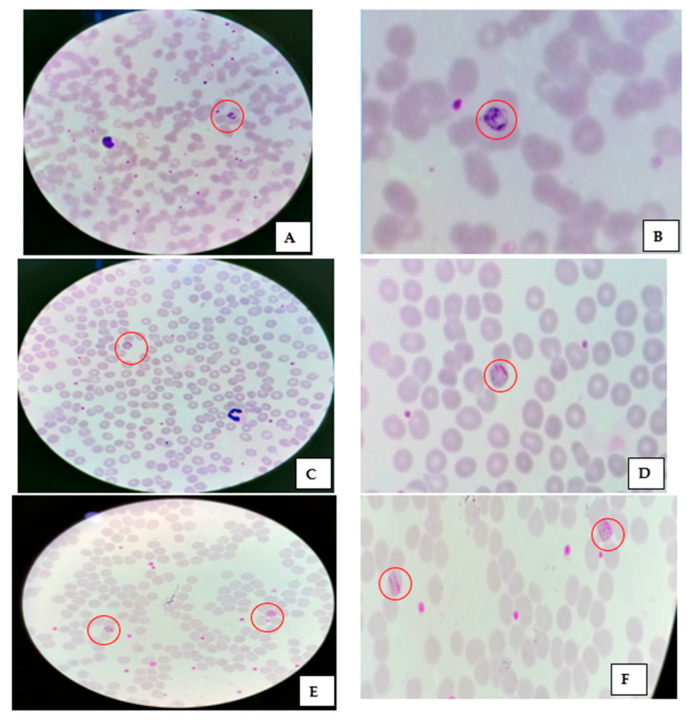
Thin smear and its magnified images showing (**A**,**B**) a developing schizont; (**C**,**D**) a gametocyte; (**E**,**F**) the band form of trophozoites.

Thick smears showed trophozoites of *P. malariae*, and their respective magnified images are depicted in Figure 3A–D. A thin smear and its magnified images of *P. malariae* are shown in Figure 4A–F. The developing schizonts, gametocytes, and band form of trophozoites were confirmed in thin smears. The *Plasmodium* parasites were differentiated as rings, trophozoites, schizonts, and male/female gametocytes based on their morphological characteristics. *P. malariae* were distinguished by the features of senescent RBC infection, band-like trophozoites, and rosette forms of merozoites which are lined up around the perimeter of a schizont. The unique part of *P. malariae* is the band/sash distinctive structure developed as the trophozoite matures. Other *Plasmodium* species also have specified characteristics, e.g., *P. falciparum* have thin and delicate ring-form trophozoites that possess one or two chromatin dots, preferably located on the periphery of RBCs and crescent-shaped gametocytes. In *P. vivax*, the infected erythrocytes are enlarged, and Schüffner’s dots appear in the cytoplasm as the rings mature into trophozoites. *P. ovale* and *P. vivax* have similar morphological features, except Schüffner’s dots are distorted into an oval shape and feathering around the edges of the RBCs.

Further analysis revealed that malaria infection is lowest among the under-5 age groups, and no significant difference was observed between the age groups. When the parasite infection was looked into, *P. malariae* and mixed infections were highest among the 5–15 years group than in other groups. However, *P. vivax* was most prevalent in the ≥15 years group. Moreover, *Plasmodium* parasite infections were not significantly different between male and female subjects (Table 1).

The overall risk analysis of malaria infection among the different age groups is shown in Table 2. The 5–15 years and ≥15 years age groups had a 1.67 (95% CI 0.94–2.94) and 1.69 (95% CI 0.99–2.87) times higher risk of malaria infection compared to the under-5 age group. The risk of *P. falciparum* infection in the 5–15 years and ≥15 years age groups were 1.99 (95% CI 1.06–3.7) and 1.82 (95% CI 1.01–3.29) times higher, respectively, and varied significantly (*p* < 0.05). Among the three age groups, the risk of non-*P. falciparum* infection was found to be highest among the ≥15 years group—1.22 (95% CI, 0.40–3.67) times higher compared to the under-5 age group (Table 2).

The mean parasite density of *P. falciparum* was highest, i.e., 12,275.08 (95% CI, 9056.38–15,493.78) compared to *P. vivax* and *P. malariae,* which were 7618.12 (95% CI, 4421.556.10–14,426.30) and 1455.00 (95% CI, 200.22–2709.77), respectively. 

## 4. Discussion

**Figure 5:**
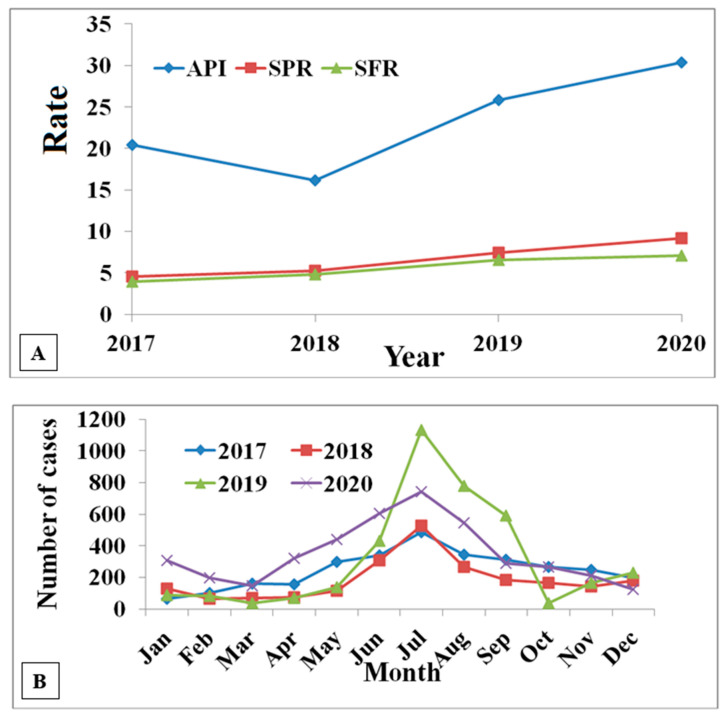
(**A**): Malariometric indices (API, SPR, SFR) in Lawngtlai District of the State of Mizoram from 2017 to 2020; (**B**) monthly total malaria cases from 2017 to 2020 (source: State Vector-Borne Diseases Control Programme, Mizoram). Note: API: annual parasite index, SPR: slide positivity rate, SFR: slide *falciparum* rate).

Malaria remains a significant public health problem in Mizoram. The hilly and mountainous terrain, variable ecological habitats, heavy rainfall, and forest-dwelling provide a fertile ground for the prevalence of different human *Plasmodium* species. *P. falciparum* is the most prevalent human malaria species in Langwatali District. In the study district, no significant difference (*p* > 0.05) in the slide positivity rate and the slide *Falciparum* rate was observed even after the high annual blood examination rate of 29.54. The annual parasite index was between 20.4 to 30.4 from 2017 to 2020 (Figure 5A). The monthly malaria data from 2017 to 2020 showed that malaria transmission was perennial (throughout the year) in Langwatali District (Figure 5B). Early diagnosis, treatment, and introduction of long-lasting insecticidal nets as vector control strategies have significantly reduced malaria incidence in the northeastern states of India. The API of Mizoram decreased from 20.71 to 6.98 (2014–2019). The proper implementation of malaria control strategies by the State Vector-Borne Disease Control Programme has successfully combatted *P. falciparum* malaria, the deadliest among human *Plasmodium* species in the hard-to-reach areas of Mizoram. 

*P. malariae* is considered a benign malaria parasite because of low parasite density and coinfections with other *Plasmodium* species. The density and infectivity of *Plasmodium* species depend on their potential for invasion into the different age stages of RBCs. The low parasite density of *P. vivax* is attributed to its invasion capacity only in reticulocytes (<1% of all erythrocytes). *P. falciparum* is efficient in invading all age stages of RBCs. However, *P. malariae* preferred to invade mature RBCs [22,23]. Thus, *P. malariae* infection is considered asymptomatic or sub-patent, and this low parasitemia level probably has substantial chronic transmission potential [24,25]. In the last two decades, the mono-infections of *P. malariae* and *P. ovale* and their mixed infections with *P. falciparum* and *P. vivax* have been reported in India and the neighboring countries (Table 3). In Myanmar, *P. knowlesi* and *P. malariae* were reported in about <3% of the tested cases [26,27], and *P. malariae* and *P. ovale* are also prevalent in Bangladesh [28,29]. In India, *P. ovale* was identified long back in Orissa [30], and *P. ovale*, as a single case, was reported for the first time from Titabor PHC, Jorhat, Assam, in 2003 [31]. Krishna et al. (2015) also reported the prevalence of *P. malariae* in many parts of India (Orissa, Chhattisgarh, Maharashtra, Madhya Pradesh, Tripura, Gujarat, and Rajasthan), and of *P. ovale* in Orissa, Jharkhand, Chhattisgarh, Madhya Pradesh, and Jharkhand. The prevalence of *P. ovale* was also reported in Jagdalpur, Chhattisgarh [32]. One published report from Mizoram indicates the presence of mixed infections of *P. falciparum* with other *Plasmodium* species [6]. 

Malaria RDTs are the diagnostic tool of choice at points of care in hard-to-access communities and the preferred option for programmatic deployment. However, low-threshold sensitivity, identification, and quantitation of the sexual/asexual stages of *Plasmodium* have given microscopy an edge over RDTs. RDTs fail to detect low-density parasitemia, deletion of *P. falciparum* histidine-rich protein 2 (HRP2) leads to false negativity, and the stable HRP2 in the blood results in false positivity. PCR is considered highly sensitive and specific for *Plasmodium* identification and quantification. However, this technique is expensive and requires regular power backup, trained human resources, high time expenditure, and has only limited application in low-resource settings or emergencies [17]. In summary, microscopy is more reliable in detecting four human *Plasmodium* species compared to RDTs. Yet, it has the limitations of skilled microscopists, quality of the blood smear, staining, and fixation. 

Thus, to target all the *Plasmodium* species infecting humans, malaria detection using microscopy should be strengthened till the deployment of a molecular tool on the community/primary health center level.

## 5. Limitations of the Study

In the present study, RDTs and microscopy were used for parasite detection. Molecular analysis was not performed due to the unavailability of blood samples. However, the availability of a WHO-certified level I microscopist strengthened the malaria diagnosis with microscopy.

## 6. Conclusions 

Early and effective diagnosis of human malaria species, including low-density infection and asymptomatic malaria, is crucial in achieving the goal of malaria elimination. Presently, RDTs are used preferably to diagnose malaria, but they have several limitations in identifying *P. falciparum*, *P vivax*, and their mixed infection. In the present study, *P. malariae* cases were detected using microscopy as well as RDTs. Microscopy is the only choice of diagnosis for non-*P. falciparum* and *P. vivax* malaria until laboratories are equipped with molecular diagnostic tools at the community and primary health center levels. Thus, it is necessary to strengthen microscopy for accurate and early diagnosis of species-specific malaria until the deployment of molecular tools [5,10,37].

## Figures and Tables

**Table 1:** Age group- and gender-wise malaria cases diagnosis using RDTs and microscopy.

Age	Number of Fever Cases: 948	Rapid Diagnostic Test, n (%)					Malaria Microscopy, n (%)				
	Gender (n)	Malaria-Positive (%)	*Pf* (%)	*Pv* (%)	*Pm* (%)	*Mix* (%)	Malaria-Positive (%)	*Pf* (%)	*Pv* (%)	*Pm* (%)	*Mix* (%)
**Under-5 years**	Male (86)	8 (9.30)	7(8.14)	1 (1.16)	0	0	9 (10.47)	7 (8.14)	2 (2.33)	0	0
	Female (64)	8 (12.50)	6(9.38)	2 (3.13)	0	0	9 (14.06)	7 (10.94)	2 (3.13)	0	0
**5–15 years**	Male (164)	22 (13.41)	21(12.80)	1 (0.61)	0	0	30 (18.29)	27 (16.46)	1 (0.61)	2 (1.22)	0
	Female (111)	22 (19.81)	22 (19.82)	0	0	0	25 (22.52)	24 (21.62)	0	0	1 (0.90)
**≥15 years**	Male (262)	55 (20.99)	49 (18.70)	6 (2.29)	0	0	65 (24.81)	55 (20.99)	7 (2.67)	1 (0.38)	2 (0.76)
	Female (261)	36 (13.79)	31 (11.88)	5 (1.92)	0	0	41 (15.71)	34 (13.03)	5 (1.92)	2 (0.77)	0 (0.00)
**Total**	Male (512)	85 (16.60)	77 (15.34)	8 (1.56)	0	0	104 (20.31)	89 (17.38)	10 (1.953)	3 (0.58)	2 (0.39)
	Female (436)	66 (15.14)	59 (13.53)	7 (1.60)	0	0	75 (17.20)	65 (14.91)	7 (1.61)	2 (0.46)	1 (0.23)

*Pf*: *P. falciparum*; *Pv*: *P. vivax*; *Pm*: *P. malariae*; *Mix* (*P. falciparum* + *P. vivax*).

**Table 2:** Odds ratio of infection of parasite species among different age groups.

Age Group	BSE	Risk of Malaria Infection		Risk of *P. Falciparum* * Infection		Risk of Non-*P. falciparum* ** Infection	
		+ve	Odds Ratio (95% CI), *p*-Value	*Pf*	Odds Ratio (95% CI), *p*-Value	Non-*Pf*	Odds Ratio (95% CI), *p*-Value
Under-5 years	150	18	–	14	–	4	–
5–15 years	275	55	1.67 (0.94–2.94), *p* = 0.078	51	1.99 (1.06–3.71), *p* = 0.03	4	0.55 (0.13 to 2.21), *p* = 0.39
≥15 years	523	106	1.69 (0.99–2.87), *p* = 0.053	89	1.82 (1.01–3.29), *p* = 0.046	17	1.22 (0.40 to 3.67), *p* = 0.73

BSE: blood smear examination, *Pf*: *Plasmodium falciparum* *, non-*P. falciparum* **: other than *Plasmodium falciparum*, i.e., *P. vivax*, *P. malariae*, and *P. falciparum* + *P. vivax*.

**Table 3:** Prevalence of *Plasmodium malariae* and other neglected *Plasmodium* species in India and the adjoining countries bordering the northeastern states.

Country	*Plasmodium* Species	Study Site	Positivity	Remarks	Reference
Myanmar	*P. knowlesi*	Southern Myanmar near Yunnan Province of China	*Pk*: 4 *Pk + Pf*: 13 *Pk + Pv*: 13 *Pk + Pf + Pv*: 2	*M. nemestrina as* reservoir host	[26]
	*P. malariae*	Ranong province border	*Pm*: 1	Both microscopy and PCR were capable to identify	[27]
Bangladesh	*P. malariae*	Chittagong Hill Tracts in southeastern Bangladesh	*Pm*: 60	*P. malariae* is highly underestimated in rural Bangladesh	[29]
	*P. malariae* and *P. ovale*	Chittagong Hill Tracts	*Pm*: 3 *Po*: 3	Both microscopy and PCR were capable to identify	[28]
India	*P. ovale*	Koraput District, Orissa state.	*Po*: 3	–	[30]
	*P. ovale*	Titabor PHC, Jorhat (Assam)	*Po*: 1	–	[31]
	*P. falciparum + P. ovale*	Orissa, Jharkhand, Chhattisgarh, Madhya Pradesh	*Pf + Po*: 6	Mixed infection	[33]
	*P. falciparum + P. malariae + P. ovale*	Jharkhand	*Pf + Pm + P*: 01	Mixed infection	[33]
	*P. falciparum + P. malariae*	Orissa, Chhattisgarh, Maharashtra, Madhya Pradesh, Tripura, Gujarat, and Rajasthan	*Pf + Pm*: 19	Mixed infection	[33]
	*P. ovale curtisi* and *P. ovale wallikeri*	Bastar, State of Chhattisgarh	*Po*: 2 *Pv* + *Pf* + *Po*: 1	Mono- and mixed infection of *P. ovale curtisi*	[34]
	*P. ovale*	Jagdalpur (Chhattisgarh)	*Po*: 3	–	[32]
	*P. malariae* (mono- and mixed infections)	Balaghat District	*Pm*: 14 *Pm* + *Pv*: 2 *Pm* + *Pf*: 3	Existence in forest villages of central India	[4]
	*P. malariae*	Lohit (Arunachal Pradesh)	*Pm*: 9	Confirmed by nested PCR	[35]
	*P. malariae*	Orissa	*Pm*: 108	High prevalence	[36]

*Pf* = *Plasmodium falciparum*; *Pv* = *P. vivax*; *Pm* = *P. malariae*; *Po* = *P. ovale*; *Pk* = *P. knowlesi*.

## Data Availability

All the data generated or analyzed during this study are included in this published article.

## Abbreviations

*Pf*: *Plasmodium falciparum*; RDTs: rapid diagnostic tests, HRP2: histidine-rich protein 2, WHO: World Health Organization, PCR: polymerase chain reaction.
